# The Role of Risk Perception and Psychosocial Factors in Attitudes and Views on Preclinical Dementia Risk Estimation – The PreTAD‐Study

**DOI:** 10.1002/alz70857_098147

**Published:** 2025-12-24

**Authors:** Michelle Gerards, Constanze Hübner, Annika Baumeister, Federica Ribaldi, Yahveth Cantero‐Fortiz, Julia Braun, Mercè Boada, Giovanni B Frisoni, Björn Schmitz‐Luhn, Carolin Schwegler, Christiane Woopen, Frank Jessen, Ayda Rostamzadeh

**Affiliations:** ^1^ Department of Psychiatry and Psychotherapy, Medical Faculty, University of Cologne, Cologne, Germany; ^2^ Center for Life Ethics, University of Bonn, Bonn, Germany; ^3^ Laboratory of Neuroimaging of Aging (LANVIE), University of Geneva, Geneva, Switzerland; ^4^ Geneva Memory Center, Department of Rehabilitation and Geriatrics, Geneva University Hospitals, Geneva, Geneva, Switzerland; ^5^ Ace Alzheimer Center Barcelona – Universitat Internacional de Catalunya, Barcelona, Spain; ^6^ Center for Life Ethics, University of Bonn, Bonn, Nrw, Germany; ^7^ Ace Alzheimer Center Barcelona‐Universitat Internacional de Catalunya, Barcelona, Spain; ^8^ Networking Research Center on Neurodegenerative Diseases (CIBERNED), Instituto de Salud Carlos III, Madrid, Spain; ^9^ Geneva Memory Center, Department of Rehabilitation and Geriatrics, Geneva University Hospitals, Geneva, Switzerland; ^10^ Center for Life Ethics, University of Bonn, Bonn, NRW, Germany; ^11^ Faculty of Arts and Humanities, University of Cologne, Cologne, Germany; ^12^ German Center for Neurodegenerative Diseases (DZNE), Bonn, Germany; ^13^ Excellence Cluster on Cellular Stress Responses in Aging‐Associated Diseases (CECAD), University of Cologne, Cologne, Germany

## Abstract

**Background:**

Advances in disease‐modifying treatments and blood‐based biomarkers for Alzheimer's disease (AD) have increased the importance of early diagnosis and dementia risk estimation before symptom onset. Autonomous decisions about AD risk estimation are complex and should align with individual needs and preferences. The PreTAD study (Predictive Turn in Alzheimer's Disease: Ethical, Clinical, Linguistic, and Legal Aspects) evaluates and compares attitudes on dementia risk estimation among first‐degree relatives of individuals with dementia and patients with subjective cognitive decline to improve counseling for preclinical predictive AD diagnostics in the future.

**Method:**

PreTAD assesses risk perception, attitudes and views on AD dementia risk estimation, along with influencing psychosocial factors (living situation, loneliness, resilience, anxiety, and depression). The risk perception survey assessed participants’ opinions on two aspects: (1) what percentage they consider to represent a high general risk of developing AD dementia (perceived high general dementia risk), and (2) their perceived personal risk of developing AD dementia within 10 years (personal dementia risk).

**Result:**

Results from 390 participants in Germany, Switzerland, and Spain are presented. The perceived high general dementia risk assessed by participants ranged from >0%‐100%. Around 30% perceived their personal dementia risk as 0–10%, while 70% rated their personal dementia risk as higher than 10%. Higher estimated personal dementia risk was associated with a greater estimated impact of biomarker‐based early dementia risk estimation and clinical trial participation on the decision to estimate one's own dementia risk. Perceived high general dementia risk correlated with a higher influence of income, belief in the impact of lifestyle changes, and preference for high test accuracy over low invasiveness on the decision to estimate dementia risk. Higher perceived personal dementia risk correlated with lower resilience and higher anxiety.

**Conclusion:**

Risk perception is highly individual and may influence the process of AD dementia risk estimation. While psychosocial factors appear to have a limited impact on the decision to pursue dementia risk estimation in a hypothetical scenario, their impact in the subsequent process remains unclear. PreTAD findings highlight the need for personalized counseling that considers emotional and cognitive profiles to support informed decision‐making in preclinical predictive AD diagnostics.

